# 571 Ethnicity and Etiology in Burn Patients for the American Burn Association National Burn Repository (NBR)

**DOI:** 10.1093/jbcr/irac012.199

**Published:** 2022-03-23

**Authors:** Ahmed M Al Hosni, Connor McSweeney, Fagun Jain, Anthony Papp

**Affiliations:** University British Columbia, Department of Plastic Surgery, Vancouver, British Columbia; University of British Columbia - Medical School, Vancouver, British Columbia; University British Columbia, Department of Plastic Surgery, Vancouver, British Columbia; University of British Columbia, Department of Plastic Surgery, Vancouver, British Columbia

## Abstract

**Introduction:**

The purpose of this study was to analyze data from the American Burn Association National Burn Repository (NBR)

with particular focus on patient ethnicity and burn etiology. We hypothesize that burn etiology, severity and other characteristics will be significantly different between differing ethnic groups throughout the database. This information can be used to augment burn prevention strategies by targeting at risk ethnic groups.

**Methods:**

Data on burn patients was derived from the American Burn Association National Burn Registry including all burn entries for a 10 year period (2009 to 2018) from 46 burn centers. The ethnic categories for this study were White, Black, Hispanic, Indigenous and Asian. The study also involved analysis of patient demographics, burn severity, context of injury, and hospital course.

**Results:**

White patients were the largest group (64.0%), had the highest proportion of flame injury (53.1%) and shared the highest mortality rate with indigenous patients (3.1% compared with 2.6% for all other ethnicities). Black individuals (22.2%) had higher rates of scald burns (53.2%), the shortest average hospital stay (16.8 days) and along with indigenous patients the highest rates of assault/abuse (2.0% and 1.9% respectively). Hispanic patients (10.0%) had more scald burns (47%), the largest proportion of men (66.5%), the highest incident of work-related injuries (18.0%) and the largest average TBSA at 10.1%. Asian patients (2.7%) had the largest proportion of scald injury (63.1%) and the smallest proportion of male patients (54.5%). Indigenous patients (1.1%) had higher rates of flame burns, suffered full thickness burns at the highest rate (32.8%), had the longest average stays in hospital (21) and the ICU (15) and had the highest rates of blanks for data entry.

**Conclusions:**

This study found multiple significant differences in burn populations when compared by ethnicity. We have found that the indigenous population suffered full thickness burns at the highest rate and have had the longest average hospital stay as well average ICU stay. We have also had the unexpected finding of higher rates of unknowns in the indigenous population which may reflect racial bias at an institutional level nationally.

